# A Pooled Analysis of Body Mass Index and Mortality among African Americans

**DOI:** 10.1371/journal.pone.0111980

**Published:** 2014-11-17

**Authors:** Sarah S. Cohen, Yikyung Park, Lisa B. Signorello, Alpa V. Patel, Deborah A. Boggs, Laurence N. Kolonel, Cari M. Kitahara, Synnove F. Knutsen, Elizabeth Gillanders, Kristine R. Monroe, Amy Berrington de Gonzalez, Traci N. Bethea, Amanda Black, Gary Fraser, Susan Gapstur, Patricia Hartge, Charles E. Matthews, Song-Yi Park, Mark P. Purdue, Pramil Singh, Chinonye Harvey, William J. Blot, Julie R. Palmer

**Affiliations:** 1 International Epidemiology Institute, Rockville, Maryland, United States of America; 2 EpidStat Institute, Ann Arbor, Michigan, United States of America; 3 Division of Cancer Epidemiology and Genetics, National Cancer Institute, National Institutes of Health, Bethesda, Maryland, United States of America; 4 Department of Epidemiology, Harvard School of Public Health, Boston, Massachusetts, United States of America; 5 Epidemiology Research Program, American Cancer Society, Atlanta, Georgia, United States of America; 6 Slone Epidemiology Center at Boston University, Boston, Massachusetts, United States of America; 7 Cancer Epidemiology Program, University of Hawaii Cancer Center, Honolulu, Hawaii, United States of America; 8 Center for Nutrition, Healthy Lifestyle and Disease Prevention, School of Public Health, Loma Linda University, Loma Linda, California, United States of America; 9 Division of Cancer Control and Population Sciences, National Cancer Institute, National Institutes of Health, Bethesda, Maryland, United States of America; 10 Department of Preventive Medicine, Keck School of Medicine, University of Southern California, Los Angeles, California, United States of America; 11 Center for Health Research, School of Public Health, Loma Linda University, Loma Linda, California, United States of America; 12 Epidemiology and Genomics Research Program, National Cancer Institute, National Institutes of Health, Bethesda, Maryland, United States of America; 13 Division of Epidemiology, Department of Medicine, Vanderbilt University Medical Center and Vanderbilt-Ingram Cancer Center, Nashville, Tennessee, United States of America; National Institute of Agronomic Research, France

## Abstract

Pooled analyses among whites and East Asians have demonstrated positive associations between all-cause mortality and body mass index (BMI), but studies of African Americans have yielded less consistent results. We examined the association between BMI and all-cause mortality in a sample of African Americans pooled from seven prospective cohort studies: NIH-AARP, 1995–2009; Adventist Health Study 2, 2002–2008; Black Women's Health Study, 1995–2009; Cancer Prevention Study II, 1982–2008; Multiethnic Cohort Study, 1993–2007; Prostate, Lung, Colorectal and Ovarian Screening Trial, 1993–2009; Southern Community Cohort Study, 2002–2009. 239,526 African Americans (including 100,175 never smokers without baseline heart disease, stroke, or cancer), age 30–104 (mean 52) and 71% female, were followed up to 26.5 years (mean 11.7). Hazard ratios (HR) and 95% confidence intervals (CI) for mortality were derived from multivariate Cox proportional hazards models. Among healthy, never smokers (11,386 deaths), HRs (CI) for BMI 25–27.4, 27.5–29.9, 30–34.9, 35–39.9, 40–49.9, and 50–60 kg/m^2^ were 1.02 (0.92–1.12), 1.06 (0.95–1.18), 1.32 (1.18–1.47), 1.54 (1.29–1.83), 1.93 (1.46–2.56), and 1.93 (0.80–4.69), respectively among men and 1.06 (0.99–1.15), 1.15 (1.06–1.25), 1.24 (1.15–1.34), 1.58 (1.43–1.74), 1.80 (1.60–2.02), and 2.31 (1.74–3.07) respectively among women (reference category 22.5–24.9). HRs were highest among those with the highest educational attainment, longest follow-up, and for cardiovascular disease mortality. Obesity was associated with a higher risk of mortality in African Americans, similar to that observed in pooled analyses of whites and East Asians. This study provides compelling evidence to support public health efforts to prevent excess weight gain and obesity in African Americans.

## Introduction

The prevalence of obesity has increased rapidly in recent decades in the United States [1] [2], and the increase has been especially pronounced in African American women where the obesity prevalence is predicted to be over 70% by 2020 [3]. Two recent large pooled cohort analyses among white adults found that obesity is associated with up to two-fold increased risks for all-cause mortality [4] [5]. Similar but somewhat weaker relative risks were also observed in a pooled analysis among East Asians [6]. However, results to date from individual studies of African Americans have been less consistent [7] [8] [9] [10] [11] [12] [13]. For this reason, we examined mortality in relation to BMI in a pooled analysis of African American participants from seven epidemiologic cohorts with appreciable numbers of African American participants.

## Methods

### Cohort Inclusion Criteria

Prospective cohort studies participating in the National Cancer Institute Cohort Consortium [14] that had at least 7,500 African American participants were eligible for inclusion. Seven cohorts were included (Table 1), in total comprising 256,409 individuals who self-reported their race as black/African American (N = 74,955 males, N = 181,443 females, and N = 11 missing gender). Of these 256,409 participants, individuals with missing age or gender (N = 44), missing BMI (N = 8,899), BMI <15 kg/m^2^ or ≥60 kg/m^2^ (N = 564) were excluded as were 7,343 participants with less than one year of follow-up and 33 who ended follow-up before age 30, leaving 239,526 participants for analysis.

**Table 1:** Descriptive characteristics of African American participants in seven cohorts included in African American BMI-Mortality Pooling Project.

	AARP		AHS2		BWHS		CPSII		MEC		PLCO		SCCS		TOTAL	
**Enrollment period**	1995–1997		2002–2007		1995		1982–1983		1993–1998		1993–2001		2002–2008			
**All eligible** a																
Participants	20,399		22,696		58,001		48,712		29,306		7,506		52,906		239,526	
Deaths	3,854		649		2,719		22,671		9,710		1,445		2,981		44,029	
Age (yrs), Mean [range] b	61 [50–71]		53 [24–104]		39 [20–70]		55 [29–90]		61 [45–78]		62 [53–77]		51 [40–79]		52.0 [20–104]	
Follow-up (yrs), Mean [range]	12.5 [14.2]		5.5 [10.0]		14.5 [14.9]		20.6 [26.5]		8.9 [16.2]		10.2 [13.0]		4.8 [7.8]		11.7 [26.5]	
**Healthy, never smokers with available covariates information** c																
Participants	6,676		15,608		34,304		19,000		7,460		675		16,452		100,175	
Deaths	788		234		868		7,507		1,436		66		487		11,386	
**Characteristics** d	**N**	**%**	**N**	**%**	**N**	**%**	**N**	**%**	**N**	**%**	**N**	**%**	**N**	**%**	**N**	**%**
**Gender**																
Male	8,782	(43)	6,795	(30)	0	(0)	17,578	(36)	10,738	(37)	3,275	(44)	22,008	(42)	69,176	(29)
Female	11,617	(57)	15,901	(70)	58,001	(100)	31,134	(64)	18,568	(63)	4,231	(56)	30,898	(58)	170,350	(71)
**Age at enrollment (yrs)**																
<40	0	(0)	3,798	(17)	30,974	(53)	3,968	(8)	0	(0)	0	(0)	0	(0)	38,740	(16)
40–49	0	(0)	6,104	(27)	16,620	(29)	11,064	(23)	3,909	(13)	0	(0)	25,695	(49)	63,392	(26)
50–59	8,652	(42)	5,868	(26)	7,349	(13)	15,929	(33)	8,181	(28)	2,651	(35)	18,064	(34)	66,694	(28)
60–69	11,262	(55)	3,945	(17)	2,995	(5)	11,659	(24)	10,464	(36)	3,882	(52)	7,025	(13)	51,232	(21)
70–79	485	(2)	2,248	(10)	63	(0)	4,906	(10)	6,752	(23)	973	(13)	2,122	(4)	17,549	(7)
80+	0	(0)	733	(3)	0	(0)	1,186	(2)	0	(0)	0	(0)	0	(0)	1,919	(1)
**Education**																
Lt High School	2,123	(11)	2,630	(12)	1,559	(3)	13,194	(28)	4,151	(14)	1,420	(19)	16,407	(31)	41,484	(18)
High School	3,938	(20)	2,558	(12)	9,557	(17)	8,777	(19)	7,655	(26)	1,560	(21)	17,890	(34)	51,935	(22)
Post High School e	1,802	(9)	1,430	(6)	0	(0)	2,954	(6)	1,768	(6)	799	(11)	2,988	(6)	11,741	(5)
Some college	5,189	(27)	7,702	(35)	20,837	(36)	8,673	(19)	8,839	(31)	1,839	(25)	10,205	(19)	63,284	(27)
College	2,941	(15)	4,146	(19)	13,695	(24)	5,256	(11)	3,410	(12)	840	(11)	3,587	(7)	33,875	(14)
> College	3,395	(18)	3,775	(17)	12,245	(21)	7,696	(17)	3,151	(11)	1,027	(14)	1,782	(3)	33,071	(14)
Missing	1,011		455		108		2,162		332		21		47		4,136	
**Marital Status**																
Married	9,767	(48)	13,265	(60)	22,899	(40)	32,263	(68)	13,547	(47)	3,799	(51)	15,348	(29)	110,888	(47)
Divorced	5,770	(29)	4,218	(19)	12,796	(22)	6,460	(14)	8,234	(29)	2,100	(28)	17,938	(34)	57,516	(24)
Widowed	3,348	(17)	1,940	(9)	2,020	(4)	6,418	(13)	5,209	(18)	1,197	(16)	5,128	(10)	25,260	(11)
Single	1,266	(6)	2,809	(13)	19,693	(34)	2,428	(5)	1,873	(6)	394	(5)	14,224	(27)	42,687	(18)
Missing	248		464		593		1,143		443		16		268		3,175	
**Alcohol (g/day)**																
None	8,050	(39)	21,521	(95)	41,140	(72)	33,297	(69)	15,667	(56)	898	(35)	23,273	(45)	143,846	(62)
< 5	7,634	(37)	817	(4)	7,302	(13)	8,038	(17)	6,221	(22)	1083	(43)	11,520	(22)	42,615	(18)
5–10	1,451	(7)	196	(1)	4,093	(7)	0	(0)	1,607	(6)	155	(6)	3,036	(6)	10,538	(5)
10–15	732	(4)	99	(0)	1,965	(3)	939	(2)	1,012	(4)	79	(3)	2,103	(4)	6,979	(3)
15–30	1,132	(6)	51	(0)	1,888	(3)	2,107	(4)	1,569	(6)	152	(6)	3,415	(7)	10,314	(4)
30+	1,400	(7)	12	(0)	1,115	(2)	3,872	(8)	1,988	(7)	177	(7)	8,586	(17)	17,150	(7)
Missing	0				498		409		1,242		4,962		973		8,084	
**Physical Activity**																
Low	11,637	(58)	13,225	(58)	18,735	(34)	12,824	(27)	6,453	(24)	525	(29)	36,809	(71)	100,208	(44)
Medium	5,181	(26)	6,625	(29)	21,794	(39)	28,625	(61)	11,750	(44)	588	(32)	6,517	(13)	81,080	(36)
High	3,167	(16)	2,846	(13)	15,280	(27)	5,295	(11)	8,620	(32)	707	(39)	8,467	(16)	44,382	(20)
Missing	414		0		2192		1,968		2,483		5,686		1,113		13,856	
**Cigarette Smoking**																
Never	7,513	(39)	17,406	(79)	37,356	(65)	20,782	(47)	10,823	(37)	3,053	(41)	19,468	(37)	116,401	(50)
Former	8,694	(45)	4,053	(18)	11,135	(19)	8,323	(19)	11,725	(41)	3,028	(40)	10,619	(20)	57,577	(25)
Current	3,070	(16)	334	(2)	9,409	(16)	13,584	(31)	6,403	(22)	1,425	(19)	22,453	(43)	56,678	(24)
Smoker, status unknown	0	(0)	360	(2)	0	(0)	1,638	(4)	0	(0)	0	(0)	0	(0)	1,998	(1)
Missing	1,122		543		101		4,385		355		0		366		6,872	
**Baseline chronic disease** f																
Yes	3,865	(20)	2,654	(12)	2,635	(5)	7,193	(15)	7,352	(25)	1,231	(16)	7,612	(15)	32,542	(14)
No	15,353	(80)	20,042	(88)	55,366	(95)	41,497	(85)	21,954	(75)	6,275	(84)	44,595	(85)	205,092	(86)
Missing	1,171						22						699			
**BMI (kg/m^2^)**																
15–18.4	125	(1)	271	(1)	944	(2)	664	(1)	283	(1)	49	(1)	600	(1)	2,936	(1)
18.5–19.9	239	(1)	512	(2)	2,250	(4)	1,273	(3)	474	(2)	89	(1)	1,220	(2)	6,057	(3)
20–22.4	1,241	(6)	2,115	(9)	8,021	(14)	5,765	(12)	2,212	(8)	449	(6)	4,467	(8)	24,270	(10)
22.5–24.9	3,047	(15)	3,778	(17)	10,998	(19)	10,126	(21)	4,918	(17)	1,058	(14)	6,788	(13)	40,713	(17)
25–27.4	4,846	(24)	4,930	(22)	11,111	(19)	12,208	(25)	6,755	(23)	1,531	(20)	8,370	(16)	49,751	(21)
27.5–29.9	3,701	(18)	3,517	(16)	7,192	(12)	7,632	(16)	5,208	(18)	1,431	(19)	7,325	(14)	36,006	(15)
30–34.9	4,616	(23)	4,493	(20)	9,612	(17)	7,926	(16)	5,981	(20)	1,761	(23)	11,680	(22)	46,069	(19)
35–39.9	1,690	(8)	1,903	(8)	4,487	(8)	2,119	(4)	2,223	(8)	747	(10)	6,667	(13)	19,836	(8)
40–49.9	810	(4)	1,047	(5)	2,953	(5)	928	(2)	1,093	(4)	345	(5)	4,938	(9)	12,114	(5)
50–60	84	(0)	130	(1)	433	(1)	71	(0)	159	(1)	46	(1)	851	2	1,774	(1)

AARP  =  NIH-AARP (formally known as the American Association of Retired Persons) Diet and Health Study; AHS2  =  Adventist Health Study 2; BWHS  =  Black Women's Health Study; CPSII  =  Cancer Prevention Study II; MEC  =  Multiethnic Cohort Study; PLCO  =  Prostate, Lung, Colorectal, and Ovarian (PLCO) Cancer Screening Trial; SCCS  =  Southern Community Cohort Study

a Population of ‘All eligible' includes 239,526 participants as follows: 256,409 participants provided by cohorts less N = 8899 missing BMI, N = 234 with BMI <15 kg/m^2^, N = 330 with BMI >60 kg/m^2^, N = 7 with missing gender, N = 37 with missing age at enrollment, N = 7343 with one year or less of follow-up, and N = 33 people who ended follow-up before age 30.

b Age at enrollment into individual cohorts.

c Population of ‘Healthy, non-smokers' includes 109,849 participants as follows: 239,526 eligible participants less 116,253 for former or current cigarette smoking, 5579 for cancer, 5731 for heart disease/heart attack, and 2114 for stroke. Covariates selected *a priori* for inclusion in multivariate models include education, physical activity, alcohol consumption, and marital status.

d Characteristics tabulated for All Eligible population.

e The BWHS did not differentiate between post high school and some college in ascertainment of educational attainment.

f Includes heart disease, heart attack, stroke, or cancer (excluding non-melanoma skin cancer).

Participants in each of the seven cohorts provided written informed consent before participating in the study. The Adventist Health Study was approved by the Loma Linda University Institutional Review Board. The Black Women's Health Study was approved by the Boston University Medical Campus Institutional Review Board. The Cancer Prevention Study II was approved by the Emory University Institutional Review Board. The Multiethnic Cohort Study was approved by the University of Hawaii Institutional Review Board and the University of Southern California Institutional Review Board. The NIH-AARP Diet and Health Study was approved by the Special Studies Institutional Review Board of the National Cancer Institute. The Prostate, Lung, Colorectal, and Ovarian Cancer Screening Trial was approved by the Special Studies Institutional Review Board of the National Cancer Institute. The Southern Community Cohort Study was approved by the Vanderbilt University Institutional Review Board, Health Sciences Committee #1 and by the Meharry Medical College Institutional Review Board.

### Study measures

Baseline data from the seven cohorts were harmonized by Westat, Inc. (Rockville, MD). BMI at cohort entry, nearly all calculated from self-reported height and weight, was categorized in predefined categories of 15–18.4, 18.5–19.9, 20–22.4, 22.5–24.9, 25–27.4, 27.5–29.9, 30–34.9, 35–39.9, 40–49.9, and 50–60 kg/m^2^. Potential confounders were harmonized as follows: education (< high school, high school, post-high school, some college, college, and > college), marital status (married, divorced/separated, widowed, single), alcohol consumption (None, <5, 5–9, 10–14, 15–30, and 30+ grams/day), cigarette smoking (never smokers, and former and current smokers categorized by packyears of exposure) and physical activity (grouped as low, medium, or high by individual cohorts and based on categories approximately equivalent to <1 hour/week, 1–3 hours/week, and 3+ hours per week of moderate and/or vigorous activity for the majority of cohorts).

### Follow-up and mortality assessment

Follow-up began one year after cohort entry in order to exclude deaths where disease-related weight change near the time of enrollment might bias the results. For individuals less than 30 years old at the time of study entry, follow-up was started at age 30. Follow-up continued until the first of date of death, end of follow-up for each cohort, or last known date alive for those lost to follow-up. The primary outcome of interest was all-cause mortality. Broad categories of cause-specific mortality were also examined, and groupings were determined from International Classification of Diseases (ICD) codes. For cardiovascular disease (CVD), codes included ICD9: 390–459, 798; ICD 10: I00–I99. For cancer, codes included ICD9: 140–239; ICD10: C00–C97. All other causes of death excluded external causes of death.

### Statistical analysis

Cox proportional hazards regression models with age as the underlying time metric and stratified by cohort were used to estimate hazard ratios (HR) and corresponding 95% confidence intervals (CI) for all-cause, CVD, and cancer mortality by categories of BMI using a referent of 22.5–24.9 kg/m^2^, similar to previous pooled analyses [5] [6]. Models were adjusted for education, marital status, alcohol consumption, and physical activity. The primary analysis was limited to individuals who self-reported no major chronic illness at baseline (heart disease, cancer excluding non-melanoma skin cancer, or stroke) and who had never smoked in order to reduce the possibility of uncontrolled confounding from cigarette smoking and preexisting disease. Prespecified secondary analyses included all participants regardless of baseline health or smoking status with additional adjustment for cigarette smoking in 9 categories (never; former/<2.4 packyears; former/2.4–8.4 packyears; former/8.5–19.9 packyears; former/20+ packyears; current/<8 packyears; current 8–14 packyears; current/15–26 packyears; current/27+ packyears, with packyear cut-offs corresponding to 25^th^, 50^th^, and 75^th^ percentiles separately among former and current smokers). Further prespecified secondary analyses also included models stratified by education status, duration of follow-up, region of the country, age, and level of physical activity. The proportional hazards assumption was evaluated by including an interaction term between BMI and follow-up time in the primary model. All Cox regression models were conducted using SAS/STAT software, version 9.3 of the SAS System for Windows (SAS Institute Inc., Cary, NC).

Heterogeneity among cohorts was assessed using the Q and I^2^ statistics calculated using Stata 12 Software (StataCorp, College Station, TX). Additionally, the impact of dropping individual cohorts was assessed by computing HRs among the remaining cohorts for 5-unit increases in continuous BMI within two strata, 15–24.9 and 25–60 kg/m^2^ to account for the non-linear relationship between BMI and all-cause mortality.

Age-standardized death rates were calculated by applying weights from the US Standard Population for 2000 to the crude death rates calculated in 5-year age-increments in our pooled population.

## Results

### Characteristics of cohort participants

Of the 239,526 black adults from seven cohorts who were eligible for this analysis, 71% were female, median age at cohort entry was 52 years, and participants were followed for an average of 11.7 years (maximum 26.5 years) (Table 1). At study entry, approximately one-third of the participants were overweight (BMI 25–29.9 kg/m^2^) and close to another third were obese (BMI ≥30 kg/m^2^). The prevalence of overweight was higher among men than women (44% versus 33%) while women were more likely than men to be obese (37% versus 24%). Half of the participants reported smoking, either formerly (25%) or currently (24%). The prevalence of current smokers declined monotonically with increasing BMI from 37% among those with BMI <18.5 kg/m^2^ to 16% among those with a BMI ≥40 kg/m^2^ (Table S1).

### BMI and All-cause Mortality

**Figure 1:**
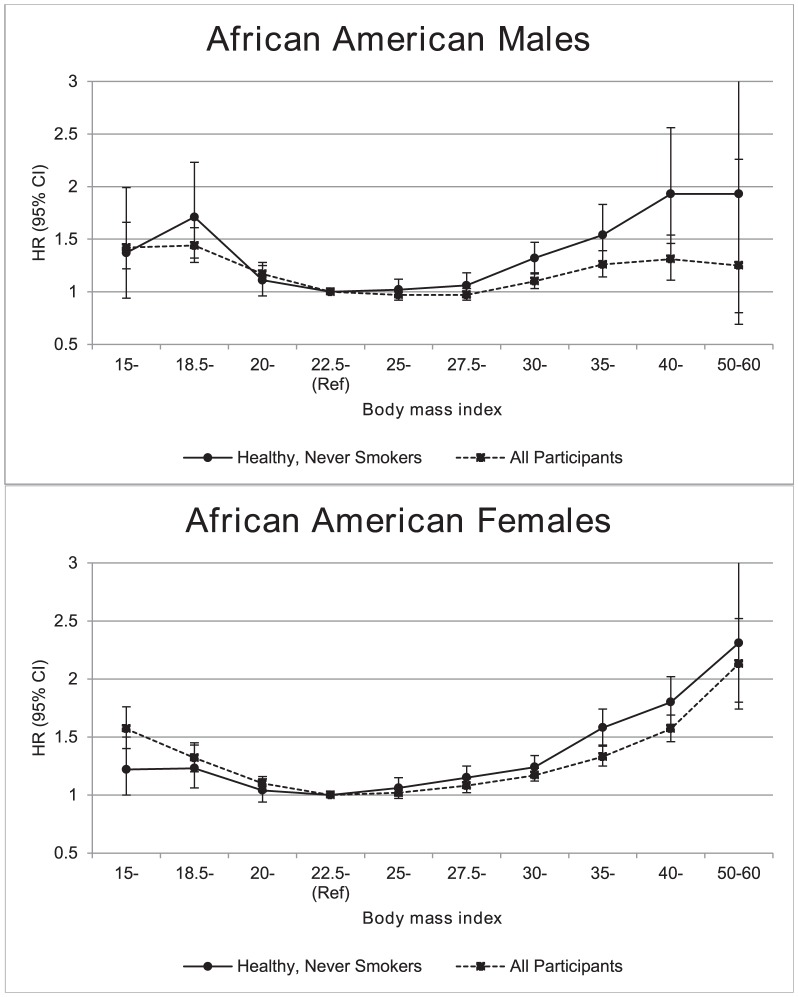
Association between body mass index and all-cause mortality, stratified by sex. Hazard ratios (HR) and 95% confidence intervals (CI) from multivariate Cox proportional hazards models for the association between body mass index (BMI) and all-cause mortality among never smokers with no baseline chronic illness (including heart disease, stroke, or cancer of any type except non-melanoma skin cancer), and among all participants, stratified by sex. NOTE: All models stratified by cohort and adjusted for education, marital status, alcohol consumption, and physical activity. Models including all participants were further adjusted for cigarette smoking status.

Among 100,175 individuals (18,060 men, 82,115 women) who were never smokers without chronic illness at baseline, 11,386 deaths occurred (3,519 in men and 7,867 in women). HRs for all-cause mortality were elevated with decreasing and increasing BMI compared to the referent of 22.5–24.9 kg/m^2^ (Table 2, Figure 1). Results were generally consistent among men and women (Table 2). HRs among women increased with progressively higher categories of BMI starting at 25–27.4 kg/m^2^, and a similar pattern was seen among men although the HRs were not significantly elevated until BMI values were ≥30 kg/m^2^. Figure 1 also displays HRs for all participants (i.e. not restricted by smoking status or chronic disease at baseline; N = 62,126 males with 16,410 deaths and N = 155,026 females with 21,297 deaths). Similar J-shaped patterns were evident in the primary, i.e. restricted, population and the non-restricted population although the magnitude of association was weaker among the non-restricted group, especially in the obese range and for men.

**Table 2:** Hazard ratios (HR) and 95% confidence intervals (CI) from multivariate Cox proportional hazards models for all-cause mortality according to categories of body mass index among African American participants without chronic illness^a^ at baseline who never smoked.

	Males				Females				Total			
	Deaths	Person Years	HR	95% CI	Deaths	Person Years	HR	95% CI	Deaths	Person Years	HR	95% CI
**BMI (kg/m^2^)**												
15–18.4	29	1,127	1.37	(0.94–1.99)	100	12,261	1.22	(1.00–1.50)	129	13,388	1.26	(1.05–1.50)
18.5–19.9	64	1,932	1.71	(1.32–2.23)	192	29,221	1.23	(1.06–1.43)	256	31,153	1.32	(1.16–1.50)
20–22.4	270	13,622	1.11	(0.96–1.28)	682	115,190	1.04	(0.94–1.14)	952	128,812	1.05	(0.98–1.14)
22.5–24.9	656	34,763	1.0	Ref	1,216	172,789	1.0	Ref	1,872	207,552	1.0	Ref
25–27.4	953	52,878	1.02	(0.92–1.12)	1,592	184,323	1.06	(0.99–1.15)	2,545	237,201	1.05	(0.98–1.11)
27.5–29.9	686	36,711	1.06	(0.95–1.18)	1,243	124,446	1.15	(1.06–1.25)	1,929	161,157	1.11	(1.05–1.19)
30–34.9	646	31,693	1.32	(1.18–1.47)	1,702	170,051	1.24	(1.15–1.34)	2,348	201,744	1.26	(1.19–1.34)
35–39.9	156	7,469	1.54	(1.29–1.83)	677	71,038	1.58	(1.43–1.74)	833	78,507	1.56	(1.43–1.69)
40–49.9	54	2,203	1.93	(1.46–2.56)	412	44,128	1.80	(1.60–2.02)	466	46,331	1.78	(1.61–1.98)
50–60	5	248	1.93	(0.80–4.69)	51	5,826	2.31	(1.74–3.07)	56	6,075	2.17	(1.66–2.83)

a Chronic illness includes heart disease, stroke, or cancer (except non-melanoma skin cancer).

Models adjusted for sex, education, marital status, alcohol consumption, and physical activity. Models stratified by cohort. Age-standardized death rates among referent BMI category (22.5-24.9) were 11.7, 6.8, and 7.8 per 1,000 person-years for males, females, and the total population, respectively. Age-standardized according to the US 2000 Standard Population using 5-year age increments.

In models stratified by duration of follow-up (Table 3), the elevated HRs for BMI categories below the referent were reduced and above the referent were increased as follow-up time increased. With 12 or more years of follow-up, no elevation in risk was seen for BMI categories below the referent while the HRs for categories above the referent were accentuated and significantly increased even at BMI 25–27.4 kg/m^2^. These patterns were also generally seen when the models were stratified by gender (Table S2). Formal tests for violation of the proportional hazards assumption in the primary analysis showed non-significant changes for males (p = 0.36) while for females the interaction (changing patterns over time) was significant (p<0.01).

**Table 3:** Hazard ratios (HR) and 95% confidence intervals (CI) from multivariate Cox proportional hazards models for all-cause mortality according to categories of body mass index among African American participants without chronic illness^a^ at baseline who never smoked, stratified by duration of follow-up and educational attainment.

	Duration of follow-up					
	Follow-up <6 years		Follow-up 6 - <12 years		Follow-up 12+ years b	
	HR	95% CI	HR	95% CI	HR	95% CI
**BMI (kg/m^2^)**						
15–18.4	1.54	(1.15–2.07)	1.27	(0.90–1.77)	1.06	(0.78–1.45)
18.5–19.9	1.56	(1.24–1.95)	1.46	(1.15–1.85)	1.03	(0.83–1.29)
20–22.4	1.17	(1.01–1.36)	1.12	(0.97–1.30)	0.96	(0.85–1.08)
22.5–24.9	1.0	Ref	1.0	Ref	1.0	Ref
25–27.4	0.92	(0.82–1.05)	1.00	(0.90–1.12)	1.14	(1.05–1.25)
27.5–29.9	0.89	(0.78–1.01)	1.10	(0.98–1.24)	1.25	(1.14–1.37)
30–34.9	1.01	(0.89–1.15)	1.26	(1.12–1.41)	1.40	(1.28–1.53)
35–39.9	1.18	(1.01–1.38)	1.47	(1.26–1.71)	1.89	(1.67–2.15)
40–60	1.41	(1.18–1.69)	1.95	(1.64–2.33)	1.92	(1.62–2.28)

	Educational attainment					
	< High School		High School		> High School	
	HR	95% CI	HR	95% CI	HR	95% CI
**BMI (kg/m^2^)**						
15–18.4	1.07	(0.78–1.46)	1.68	(1.11–2.55)	1.28	(0.99–1.66)
18.5–19.9	1.33	(1.05–1.68)	1.18	(0.87–1.60)	1.38	(1.14–1.66)
20–22.4	1.08	(0.93–1.26)	1.15	(0.96–1.37)	1.01	(0.91–1.12)
22.5–24.9	1.0	Ref	1.0	Ref	1.0	Ref
25–27.4	1.10	(0.99–1.23)	0.98	(0.86–1.12)	1.03	(0.95–1.12)
27.5–29.9	1.08	(0.96–1.21)	1.03	(0.90–1.19)	1.17	(1.07–1.28)
30–34.9	1.18	(1.06–1.32)	1.13	(0.99–1.29)	1.37	(1.26–1.50)
35–39.9	1.53	(1.32–1.77)	1.38	(1.15–1.65)	1.65	(1.46–1.86)
40–60	1.40	(1.16–1.69)	1.83	(1.50–2.23)	2.15	(1.85–2.49)

a Chronic illness includes heart disease, stroke, or cancer (except non-melanoma skin cancer).

b Participants from only 4 cohorts with duration of follow-up 12+ years (AARP, BWHS, CPSII, and MEC).

Models adjusted for sex, education, marital status, alcohol consumption, and physical activity. Models stratified by cohort. Age-adjusted death rates among referent BMI category (22.5–24.9) were 7.1, 10.1, and 9.4 per 1,000 person-years for strata of follow-up of <6 years, -<12 years, and 12+ years, respectively. Age-standardized death rates among referent BMI category (22.5–24.9) were 11.7, 9.4, and 6.9 per 1,000 person-years for strata of < high school, high school, and greater than high school education, respectively. Age-standardized according to the US 2000 Standard Population using 5-year age increments.

The association between BMI and all-cause mortality varied across levels of education. For individuals with more than a high school education, the HR in the highest category of BMI (40–60 kg/m^2^) was 2.15 (95% CI 1.85–2.49) compared to 1.40 (95% CI 1.16–1.69) for those with less than a high school education (Table 3); similar variation by education was observed among males and females separately (Table S3). Importantly, absolute death rates differed by educational category: the age-adjusted death rates for those in the referent BMI category were 11.7, 9.4, and 6.9 per 1,000 person-years for those less than 12 years of education, 12 years of education, or education beyond high school, respectively. However, similar to the excess relative risk, the excess absolute risk associated with high BMI (40–60 kg/m^2^) versus BMI of 22.5–24.9 of 7.9 per 1,000 person-years among those with more than a high school education was greater than the excess of 4.7 per 1,000 person years associated with high BMI among those with less than a high school education.

Patterns of association by region of the country were generally similar (Table S4). Results were also similar for categories of age at baseline of <40, 40–49, 50–59, and 60–69, but an association between BMI and all-cause mortality was not observed for those 70+ years of age at enrollment (Table S5). Risk of mortality increased with increasing BMI at all levels of physical activity and the associations were somewhat stronger among the most physically active (Table S6).

### BMI and Cause-specific Mortality

CVD was the leading cause of death among cohort participants (43% and 40% of deaths were due to CVD among men and women, respectively, while 25% and 27% were due to cancer). Significantly elevated HRs for CVD were evident beginning at BMI values of 25–27.4 kg/m^2^ and increased monotonically to 2.27 (95% CI 1.94–2.66) among those with BMI of 40–60 kg/m^2^ (Table 4) with similar results for men and women (Table S7).

**Table 4:** Hazard ratios (HR) and 95% confidence intervals (CI) from multivariate Cox proportional hazards models for all-cause mortality according to categories of body mass index among African American participants without chronic illness^a^ at baseline who never smoked, stratified by specific cause of death.

	Cause of Death								
	All Cancer			All CVD			All Other Non-External Causes b		
	Deaths	HR	95% CI	Deaths	HR	95% CI	Deaths	HR	95% CI
**BMI (kg/m^2^)**									
15–18.4	27	1.09	(0.74–1.60)	50	1.24	(0.93–1.66)	44	1.36	(1.00–1.85)
18.5–19.9	53	1.04	(0.78–1.37)	95	1.32	(1.06–1.63)	95	1.57	(1.26–1.95)
20–22.4	260	1.04	(0.90–1.21)	329	0.99	(0.86–1.12)	293	1.08	(0.94–1.25)
22.5–24.9	525	1.0	Ref	703	1.0	Ref	550	1.0	Ref
25–27.4	690	1.00	(0.89–1.12)	1,044	1.14	(1.04–1.25)	688	0.97	(0.87–1.09)
27.5–29.9	525	1.07	(0.95–1.21)	819	1.25	(1.13–1.39)	485	0.97	(0.86–1.09)
30–34.9	617	1.13	(1.00–1.27)	991	1.46	(1.32–1.61)	628	1.16	(1.03–1.30)
35–39.9	201	1.20	(1.01–1.41)	351	1.92	(1.69–2.19)	236	1.51	(1.30–1.77)
40–60	112	1.23	(1.00–1.51)	214	2.27	(1.94–2.66)	172	1.97	(1.65–2.36)

a Chronic illness includes heart disease, stroke, or cancer (except non-melanoma skin cancer).

b Includes all causes of death except cancer, CVD, and external causes.1.

Models adjusted for sex, education, marital status, alcohol consumption, and physical activity. Models stratified by cohort. Age-standardized death rates among referent BMI category (22.5–24.9) were 2.1, 2.9, and 2.4 per 1,000 person-years for all cancer, all CVD, and all other non-external causes of death, respectively. Age-standardized according to the US 2000 Standard Population using 5-year age increments.

HRs for cancer deaths were only modestly elevated for individuals of high BMI, with 13–23% increases for BMI ≥30 kg/m^2^ (Table 4).

### Heterogeneity across cohorts

Significant heterogeneity across the cohorts for the BMI-mortality association was observed for males (Q statistic p<0.0001) and females (p<0.0001) (Figure S1). Among males, heterogeneity across cohorts was less for BMI of 15–24.9 kg/m^2^ (p = 0.13; I^2^ = 44%) than for BMI 25–60 kg/m^2^ (p = 0.02; I^2^ = 63%) while the opposite was seen in women (for BMI 15–24.9 kg/m^2^, p = 0.01 and I^2^ = 65% versus p = 0.11 and I^2^ = 42% for BMI 25–60 kg/m^2^). Changes in HRs for 5-unit increases in continuous BMI after dropping each cohort in turn were generally negligible (Table S8). The exception was in the BMI 15–24.9 kg/m^2^ category for both men and women, where the decline in risk with rising BMI over this range was greater after dropping the CPS II cohort.

## Discussion

In this large pooled analysis of African Americans from seven prospective studies, BMI was associated with all-cause mortality risk in a J-shaped relation among never smokers without major chronic illness at study entry. With the reference category of 22.5–24.9 kg/m^2^, HRs tended to rise with decreasing BMI, reaching a 30% increased risk among the underweight. With increasing BMI, all-cause mortality risk gradually increased beginning at all levels of overweight (for women) and obesity (for men and women) and culminated in a doubling of risk among those with BMI exceeding 40 kg/m^2^. When assessed by duration of follow-up, the excess risk among the underweight was attenuated, whereas the elevated risk associated with higher levels of BMI was accentuated. Similarly shaped patterns of association between BMI and all-cause mortality were seen when all participants, including smokers and those with prevalent serious diseases, were evaluated. However, the magnitudes of the HRs were uniformly lower compared with the primary analysis of healthy, never smokers, possibly related to residual confounding from our inability to completely control for the effects of smoking and prior illness on both BMI and mortality.

Positive associations between BMI and all-cause mortality in African Americans have been reported in several large studies [7] [8] [11] [13], while other studies have yielded weaker or null results for African Americans [9] [12] [15]. Such inconsistencies were noted across published findings in several of the individual cohorts pooled here, and indeed, such differences in part prompted the current investigation. For example, in the Black Women's Health Study [11], with an average of 12.3 years of follow-up, there was a significant positive association between increased BMI and mortality, whereas in the Southern Community Cohort Study [12], with an average of 5.2 years of follow-up, being obese at cohort entry was not associated with elevated mortality among black men or women. The pooled data presented here from all seven cohorts, with stratification by length of follow-up, demonstrate the importance of longer follow-up from the time of BMI assessment, and explain, at least in part, the different findings of previous single-cohort analyses.

The observed positive association between BMI and all-cause mortality varied across strata of educational attainment, with stronger associations in the most educated group, a finding that has been observed in individual studies of African Americans [10] [11] [12]. This is not surprising given that the absolute death rate was considerably lower in the most highly educated group, although the stronger effect among the more highly educated persisted regardless of whether relative or arithmetic excess risks were considered. The link between low socioeconomic status, for which education is a close proxy, and increased risk of mortality is well-established [16] [17]. Factors such as less access to and poorer quality of care, psychosocial stress, and adverse neighborhood environment which are more common among low-income populations may compete with obesity in adversely affecting health and thus reduce the relative impact of obesity on all-cause mortality [18] [19]. Previous conflicting findings in single cohort studies of African Americans may be due in part to the different underlying populations (i.e., higher or lower socioeconomic status) in each study. At the extremes are the Black Women's Health Study with a high proportion of high school graduates and the Southern Community Cohort Study with a significant proportion of participants who had not completed high school.

In the present analysis, HRs were elevated for underweight African Americans after excluding the first year of follow-up and restricting the main analysis to individuals who didn't report heart disease, stroke, or cancer at cohort enrollment. However, as preexisting disease is strongly related to both lower BMI and higher risk of death, bias related to preexisting disease remains a plausible explanation for the increased HRs observed for low BMI. Indeed, with longer duration of follow-up, the elevated HR among the underweight disappeared.

The J-shape of the BMI and all-cause mortality association and the similarity of findings for men and women in this pooled analysis of African Americans are generally similar to the recent pooled analysis of over 1.4 million white adults [5], although the magnitude of the hazard ratios were somewhat lower in the present study than in the study of whites. Our results were also qualitatively similar to results from a large pooled analysis of Asians [6].

A recent meta-analysis examining BMI and all-cause mortality in 97 studies reported lower all-cause mortality for the overweight (BMI 25.0–29.9 kg/m^2^) than those of normal weight (BMI 18.5–24.9 kg/m^2^) with an HR of 0.94 [95% CI 0.91–0.96]) [20]. When we grouped BMI in the same, wider categories as used in the meta-analysis, our HR for overweight was 0.98 (0.90–1.06) in males and 1.07 (1.01–1.13) in females which was again not consistent with a reduced risk of mortality among the overweight. In comparing these studies, it should be noted that pooled analyses such as this one have the advantage of better control for confounding than meta-analyses, particularly for important confounders such as smoking.

Death from CVD showed the strongest association with BMI in this study with elevated HRs starting at BMI values of 25–27.5 kg/m^2^ (HR = 1.14 [95% CI 1.04–1.25]) and steadily increasing to 2.27 (95% CI 1.94–2.66) in the highest category of BMI. The strength of the association between BMI and CVD seems likely to follow from the well-established links between obesity and important cardiovascular disease risk factors such as dyslipidemia, hypertension, and insulin resistance [21]. Whether differences in the association between these risk factors and obesity vary by race remains to be clarified, but if they are present, they may contribute to differences observed in the magnitude of association by race.

In contrast to CVD mortality, the association we observed between BMI and all-cancer mortality was weaker. Even for individuals with BMI ≥35 kg/m^2^, there was only a modest elevated mortality risk (∼20%), a finding that may be explained by inverse associations with obesity for some types of cancer and positive associations with obesity for others [22].

A major strength of this pooled analysis is the very large sample of African Americans, allowing for the most precise estimates yet available of the risk of mortality in association with BMI across gender groups and over wide ranges of age and body size. To date, many fewer studies have examined this question in African Americans than in whites, and the sample sizes of the pooled analyses reflect this disparity (1.46 million whites [5] versus 239,000 African Americans); nevertheless, this large pooled analysis addresses the important question of whether previously observed increases in mortality risk associated with increased body size among white populations extend to African Americans. Additional strengths of this pooled analysis include consistent harmonization of confounders, the ability to evaluate the BMI-mortality association across a number of potentially important effect modifiers, and the long duration of follow-up.

This study was limited first by reliance on self-reported data for height and weight. There is evidence that under-reporting of weight and over-reporting of height results in systematically lower BMI values from self-report compared to measured values [23] and that this underestimation may bias associations between BMI and mortality [24]. However, substantial agreement between self-reported and measured BMI when categorized (as we have done in this analysis) has been reported [25]. Nevertheless, these results should be interpreted in light of the fact that the BMI values were derived from self-report. An additional limitation is the reliance on a single measurement in time for body size as well as chronic illness. A further limitation of this pooled analysis, is between-study heterogeneity that cannot be explained. Evidence of moderate between-study heterogeneity was noted based on relatively high I^2^ statistics, although our analyses dropping each study in turn revealed that much of the variation was related to lack of strong declining trends in risk across the 15–24.9 kg/m^2^ range in the largest cohort (CPSII).

In conclusion, this large pooled analysis demonstrated that BMI is clearly related to risk of death among African American adults, with sizeable excesses in mortality among those in the highest BMI categories. The pattern of association was similar to that observed in pooled analyses of whites and East Asians. This study provides compelling evidence to support public health efforts to prevent excess weight gain and obesity in African Americans.

## Supporting Information

Figure S1 **Forest plots examining heterogeneity across cohorts by gender and BMI (15–24.9 and 25–60 kg/m2).** Hazard ratios (and 95% confidence intervals) shown for all-cause mortality per 5-unit increase in body mass index (BMI) among healthy, never smokers.
(DOCX)

Table S1 **Prevalence of confounders by categories of body mass index.**
(DOCX)

Table S2 **Hazard ratios (HR) and 95% confidence intervals (CI) from multivariate Cox proportional hazards models for all-cause mortality according to categories of body mass index among African American participants without chronic illness at baseline who never smoked, stratified by duration of follow-up and gender.**
(DOCX)

Table S3 **Hazard ratios (HR) and 95% confidence intervals (CI) from multivariate Cox proportional hazards models for all-cause mortality according to categories of body mass index among African American participants without chronic illness at baseline who never smoked, stratified by education status and gender.**
(DOCX)

Table S4 **Hazard ratios (HR) and 95% confidence intervals (CI) from multivariate Cox proportional hazards models for all-cause mortality according to categories of body mass index among African Americans study participants without chronic illness at baseline who never smoked, stratified by region of the country.**
(DOCX)

Table S5 **Hazard ratios (HR) and 95% confidence intervals (CI) from multivariate Cox proportional hazards models for all-cause mortality according to categories of body mass index among African American participants without chronic illness at baseline who never smoked, stratified by age at baseline.**
(DOCX)

Table S6 **Hazard ratios (HR) and 95% confidence intervals (CI) from multivariate Cox proportional hazards models for all-cause mortality according to categories of body mass index among African American participants without chronic illness at baseline who never smoked, stratified by physical activity.**
(DOCX)

Table S7 **Hazard ratios (HR) and 95% confidence intervals (CI) from multivariate Cox proportional hazards models for all-cause mortality according to categories of body mass index among African American participants without chronic illness at baseline who never smoked, stratified by specific cause of death and gender.**
(DOCX)

Table S8 **Impact of omitting each cohort from analysis on hazard ratios (HR) and 95% confidence intervals (CI) for all-cause mortality per 5-unit increase in body mass index (BMI) in healthy, never smokers.**
(DOCX)
