# Supplementary material for: A Pooled Analysis of Body Mass Index and Mortality among African Americans
Source: PLoS One. 2014 Nov 17;9(11):e111980. doi: 10.1371/journal.pone.0111980 (PMC4234271; doi:10.1371/journal.pone.0111980)
Supplement: Figure S1 — Forest plots examining heterogeneity across cohorts by gender and BMI (15–24.9 and 25–60 kg/m2). Hazard ratios (and 95% confidence intervals) shown for all-cause mortality per 5-unit increase in body mass index (BMI) among healthy, never smokers. (DOCX) [file pone.0111980.s001.docx]

**Figure S1.** Forest plots examining heterogeneity across cohorts by gender and BMI (15-24.9 and 25-60 kg/m2). Hazard ratios (and 95% confidence intervals) shown for all-cause mortality per 5-unit increase in body mass index (BMI) among healthy, never smokers.

1. Females ^a^ b) Males


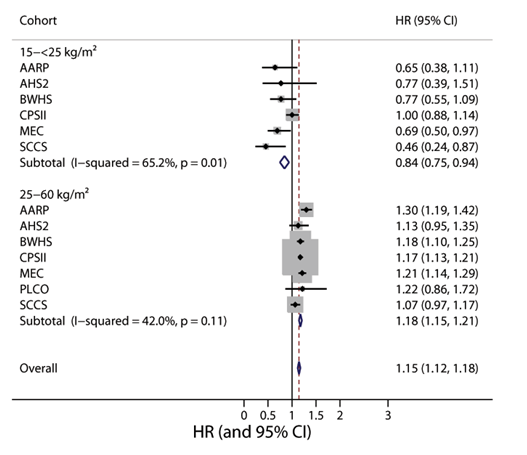

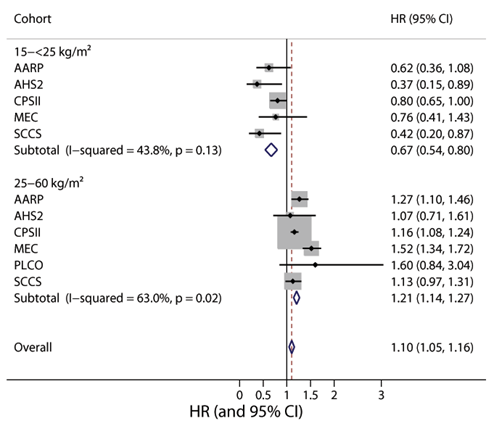


^a^ There were too few deaths in the BMI strata 15-<25 kg/m^2^ in the PLCO cohort for the statistical models to converge.

Note: Models stratified by BMI 15-24.9 and BMI 25-60 kg/m^2^. BMI analyzed as continuous measure in each stratified model. Models adjusted for education, marital status, alcohol consumption, and physical activity.
