# Supplementary material for: A Pooled Analysis of Body Mass Index and Mortality among African Americans
Source: PLoS One. 2014 Nov 17;9(11):e111980. doi: 10.1371/journal.pone.0111980 (PMC4234271; doi:10.1371/journal.pone.0111980)
Supplement: Table S1 — Prevalence of confounders by categories of body mass index. (DOCX) [file pone.0111980.s002.docx]

**Table S1.** Prevalence of confounders by categories of body mass index

|  | **Body Mass Index (kg/m^2^)** | | | | | | | | |  |
| --- | --- | --- | --- | --- | --- | --- | --- | --- | --- | --- |
|  | **15-18.4** | **18.5-19.9** | **20-22.4** | **22.5-24.9** | **25-27.4** | **27.5-29.9** | **30-34.9** | **35-39.9** | **40-60** | **TOTAL** |
| **Males** |  |  |  |  |  |  |  |  |  |  |
| All | 1% | 2% | 10% | 19% | 26% | 18% | 17% | 5% | 2% | 100% |
| Healthy, Never smokers | 1% | 1% | 8% | 18% | 27% | 19% | 19% | 6% | 2% | 100% |
| **Females** |  |  |  |  |  |  |  |  |  |  |
| All | 1% | 3% | 10% | 16% | 19% | 14% | 20% | 10% | 7% | 100% |
| Healthy, Never smokers | 1% | 3% | 11% | 17% | 19% | 14% | 19% | 9% | 7% | 100% |
| **Smoking** |  |  |  |  |  |  |  |  |  |  |
| Never | 48% | 51% | 50% | 49% | 48% | 49% | 51% | 54% | 56% | 50% |
| Former | 14% | 14% | 17% | 22% | 26% | 28% | 28% | 28% | 28% | 25% |
| Current | 37% | 35% | 32% | 28% | 25% | 22% | 20% | 18% | 16% | 24% |
| **Education** |  |  |  |  |  |  |  |  |  |  |
| Lt High School | 20% | 16% | 15% | 15% | 17% | 18% | 19% | 21% | 21% | 18% |
| High School | 20% | 21% | 19% | 20% | 21% | 23% | 24% | 25% | 26% | 22% |
| Post High School | 4% | 3% | 4% | 5% | 5% | 5% | 5% | 5% | 5% | 5% |
| Some college | 27% | 25% | 26% | 27% | 27% | 27% | 27% | 27% | 28% | 27% |
| College | 16% | 19% | 18% | 16% | 15% | 13% | 13% | 12% | 11% | 14% |
| > College | 13% | 16% | 17% | 17% | 15% | 14% | 12% | 10% | 8% | 14% |
| **Physical Activity** |  |  |  |  |  |  |  |  |  |  |
| Low | 47% | 42% | 37% | 36% | 39% | 43% | 50% | 58% | 66% | 44% |
| Medium | 36% | 36% | 39% | 40% | 39% | 37% | 33% | 28% | 23% | 36% |
| High | 17% | 22% | 24% | 23% | 22% | 20% | 17% | 14% | 11% | 20% |
| **Alcohol (g/day)** |  |  |  |  |  |  |  |  |  |  |
| None | 61% | 61% | 59% | 59% | 60% | 62% | 65% | 68% | 70% | 62% |
| < 5 | 15% | 15% | 17% | 18% | 19% | 19% | 19% | 18% | 18% | 18% |
| 5-10 | 5% | 5% | 5% | 5% | 5% | 5% | 4% | 4% | 4% | 5% |
| 10-15 | 3% | 3% | 3% | 3% | 3% | 3% | 3% | 2% | 2% | 3% |
| 15-30 | 5% | 6% | 5% | 5% | 5% | 5% | 4% | 3% | 3% | 4% |
| 30+ | 11% | 11% | 10% | 9% | 8% | 7% | 6% | 5% | 4% | 7% |
| **Marital Status** |  |  |  |  |  |  |  |  |  |  |
| Married | 34% | 37% | 43% | 49% | 52% | 51% | 47% | 40% | 34% | 47% |
| Divorced | 22% | 22% | 23% | 23% | 23% | 23% | 26% | 27% | 30% | 24% |
| Widowed | 10% | 9% | 8% | 9% | 10% | 11% | 12% | 13% | 12% | 11% |
| Single | 34% | 32% | 25% | 18% | 14% | 14% | 16% | 20% | 24% | 18% |
| **Baseline co-morbidities** |  |  |  |  |  |  |  |  |  |  |
| Heart disease/heart attack | 5% | 4% | 4% | 5% | 6% | 7% | 7% | 7% | 7% | 6% |
| Stroke | 3% | 3% | 2% | 3% | 3% | 3% | 4% | 5% | 5% | 3% |
| Cancer | 6% | 4% | 5% | 5% | 5% | 6% | 6% | 6% | 5% | 5% |
