# Supplementary material for: A Pooled Analysis of Body Mass Index and Mortality among African Americans
Source: PLoS One. 2014 Nov 17;9(11):e111980. doi: 10.1371/journal.pone.0111980 (PMC4234271; doi:10.1371/journal.pone.0111980)
Supplement: Table S2 — Hazard ratios (HR) and 95% confidence intervals (CI) from multivariate Cox proportional hazards models for all-cause mortality according to categories of body mass index among African American participants without chronic illness at baseline who never smoked, stratified by duration of follow-up and gender. (DOCX) [file pone.0111980.s003.docx]

**Table S2.** Hazard ratios (HR) and 95% confidence intervals (CI) from multivariate Cox proportional hazards models for all-cause mortality according to categories of body mass index among African American participants without chronic illness^a^ at baseline who never smoked, stratified by duration of follow-up and gender.

| **MALES** | **Duration of follow-up** | | | | | | | | |
| --- | --- | --- | --- | --- | --- | --- | --- | --- | --- |
|  | **Follow-up < 6 years** | | | **Follow-up 6 - <12 years** | | | **Follow-up 12+ years** ^b^ | | |
|  | **HR** | **95% CI** | | **HR** | **95% CI** | | **HR** | **95% CI** | |
| **BMI (kg/m^2^)** |  |  |  |  |  |  |  |  |  |
| 15-18.4 | 1.71 | (0.99- | 2.96) | 1.63 | (0.79- | 3.35) | 0.93 | (0.44- | 1.98) |
| 18.5-19.9 | 1.82 | (1.22- | 2.71) | 1.72 | (1.03- | 2.88) | 1.60 | (0.98- | 2.62) |
| 20-22.4 | 0.98 | (0.75- | 1.28) | 1.51 | (1.17- | 1.94) | 0.93 | (0.74- | 1.18) |
| 22.5-24.9 | 1.0 | Ref |  | 1.0 | Ref |  | 1.0 | Ref |  |
| 25-27.4 | 0.92 | (0.76- | 1.12) | 1.02 | (0.85- | 1.24) | 1.08 | (0.93- | 1.26) |
| 27.5-29.9 | 0.75 | (0.60- | 0.94) | 1.30 | (1.07- | 1.59) | 1.14 | (0.97- | 1.34) |
| 30-34.9 | 0.96 | (0.78- | 1.20) | 1.55 | (1.26- | 1.90) | 1.43 | (1.21- | 1.69) |
| 35-39.9 | 1.08 | (0.78- | 1.48) | 1.59 | (1.14- | 2.21) | 1.93 | (1.46- | 2.56) |
| 40-60 | 1.42 | (0.92- | 2.17) | 2.85 | (1.78- | 4.57) | 1.68 | (0.98- | 2.89) |
| **FEMALES** | **Duration of follow-up** | | | | | | | | |
|  | **Follow-up < 6 years** | | | **Follow-up 6 - <12 years** | | | **Follow-up 12+ years** ^b^ | | |
|  | **HR** | **95% CI** | | **HR** | **95% CI** | | **HR** | **95% CI** | |
| **BMI (kg/m^2^)** |  |  |  |  |  |  |  |  |  |
| 15-18.4 | 1.51 | (1.07- | 2.15) | 1.16 | (0.79- | 1.69) | 1.10 | (0.78- | 1.55) |
| 18.5-19.9 | 1.47 | (1.12- | 1.94) | 1.40 | (1.06- | 1.83) | 0.96 | (0.74- | 1.23) |
| 20-22.4 | 1.28 | (1.06- | 1.53) | 0.99 | (0.83- | 1.18) | 0.97 | (0.84- | 1.11) |
| 22.5-24.9 | 1.0 | Ref |  | 1.0 | Ref |  | 1.0 | (Ref |  |
| 25-27.4 | 0.93 | (0.79- | 1.10) | 1.00 | (0.87- | 1.15) | 1.18 | (1.06- | 1.31) |
| 27.5-29.9 | 0.99 | (0.83- | 1.17) | 1.02 | (0.88- | 1.18) | 1.32 | (1.18- | 1.48) |
| 30-34.9 | 1.06 | (0.91- | 1.24) | 1.15 | (1.00- | 1.32) | 1.39 | (1.25- | 1.55) |
| 35-39.9 | 1.27 | (1.05- | 1.53) | 1.39 | (1.17- | 1.65) | 1.92 | (1.65- | 2.22) |
| 40-60 | 1.51 | (1.23- | 1.86) | 1.79 | (1.48- | 2.17) | 1.98 | (1.65- | 2.37) |

^a^ Chronic illness includes heart disease, stroke, or cancer (except non-melanoma skin cancer)

^b^ Participants from only 4 cohorts with duration of follow-up 12+ years (AARP, BWHS, CPSII, and MEC)

Models adjusted for sex, education, marital status, alcohol consumption, and physical activity. Models stratified by cohort.
