# Supplementary material for: A Pooled Analysis of Body Mass Index and Mortality among African Americans
Source: PLoS One. 2014 Nov 17;9(11):e111980. doi: 10.1371/journal.pone.0111980 (PMC4234271; doi:10.1371/journal.pone.0111980)
Supplement: Table S3 — Hazard ratios (HR) and 95% confidence intervals (CI) from multivariate Cox proportional hazards models for all-cause mortality according to categories of body mass index among African American participants without chronic illness at baseline who never smoked, stratified by education status and gender. (DOCX) [file pone.0111980.s004.docx]

**Table S3.** Hazard ratios (HR) and 95% confidence intervals (CI) from multivariate Cox proportional hazards models for all-cause mortality according to categories of body mass index among African American participants without chronic illness^a^ at baseline who never smoked, stratified by education status and gender.

| **MALES** | **Educational attainment** | | | | | | | | |
| --- | --- | --- | --- | --- | --- | --- | --- | --- | --- |
|  | **< High School** | | | **High School** | | | **> High School** | | |
|  | **HR** | **95% CI** | | **HR** | **95% CI** | | **HR** | **95% CI** | |
| **BMI (kg/m^2^)** |  |  |  |  |  |  |  |  |  |
| 15-18.4 | 1.04 | (0.58- | 1.87) | 2.22 | (0.69- | 7.14) | 1.80 | (1.05- | 3.08) |
| 18.5-19.9 | 1.52 | (1.01- | 2.28) | 1.43 | (0.58- | 3.54) | 2.30 | (1.58- | 3.35) |
| 20-22.4 | 1.08 | (0.85- | 1.38) | 0.87 | (0.60- | 1.25) | 1.20 | (0.98- | 1.47) |
| 22.5-24.9 | 1.0 | Ref |  | 1.0 | Ref |  | 1.0 | Ref |  |
| 25-27.4 | 1.03 | (0.86- | 1.24) | 0.97 | (0.77- | 1.22) | 1.03 | (0.90- | 1.19) |
| 27.5-29.9 | 1.05 | (0.87- | 1.27) | 0.92 | (0.71- | 1.20) | 1.15 | (0.99- | 1.34) |
| 30-34.9 | 1.16 | (0.96- | 1.41) | 1.19 | (0.92- | 1.55) | 1.50 | (1.28- | 1.75) |
| 35-39.9 | 1.37 | (1.02- | 1.83) | 1.09 | (0.71- | 1.66) | 1.96 | (1.50- | 2.56) |
| 40-60 | 1.35 | (0.83- | 2.17) | 2.42 | (1.43- | 4.11) | 2.19 | (1.42- | 3.37) |
| **FEMALES** | **Educational attainment** | | | | | | | | |
|  | **< High School** | | | **High School** | | | **> High School** | | |
|  | **HR** | **95% CI** | | **HR** | **95% CI** | | **HR** | **95% CI** | |
| **BMI (kg/m^2^)** |  |  |  |  |  |  |  |  |  |
| 15-18.4 | 1.10 | (0.75- | 1.60) | 1.68 | (1.07- | 2.64) | 1.14 | (0.85- | 1.53) |
| 18.5-19.9 | 1.33 | (0.99- | 1.78) | 1.17 | (0.85- | 1.63) | 1.20 | (0.97- | 1.48) |
| 20-22.4 | 1.08 | (0.89- | 1.30) | 1.27 | (1.04- | 1.56) | 0.94 | (0.83- | 1.07) |
| 22.5-24.9 | 1.0 | Ref |  | 1.0 | Ref |  | 1.0 | Ref |  |
| 25-27.4 | 1.15 | (1.00- | 1.32) | 0.98 | (0.84- | 1.16) | 1.04 | (0.94- | 1.16) |
| 27.5-29.9 | 1.11 | (0.95- | 1.29) | 1.11 | (0.93- | 1.32) | 1.19 | (1.06- | 1.33) |
| 30-34.9 | 1.20 | (1.05- | 1.38) | 1.13 | (0.96- | 1.32) | 1.32 | (1.19- | 1.47) |
| 35-39.9 | 1.61 | (1.35- | 1.92) | 1.46 | (1.19- | 1.79) | 1.59 | (1.39- | 1.83) |
| 40-60 | 1.47 | (1.19- | 1.81) | 1.82 | (1.47- | 2.26) | 2.15 | (1.83- | 2.53) |

^a^ Chronic illness includes heart disease, stroke, or cancer (except non-melanoma skin cancer)

Models adjusted for sex, marital status, alcohol consumption, and physical activity. Models stratified by cohort.
