# Supplementary material for: A Pooled Analysis of Body Mass Index and Mortality among African Americans
Source: PLoS One. 2014 Nov 17;9(11):e111980. doi: 10.1371/journal.pone.0111980 (PMC4234271; doi:10.1371/journal.pone.0111980)
Supplement: Table S4 — Hazard ratios (HR) and 95% confidence intervals (CI) from multivariate Cox proportional hazards models for all-cause mortality according to categories of body mass index among African Americans study participants without chronic illness at baseline who never smoked, stratified by region of the country. (DOCX) [file pone.0111980.s005.docx]

**Table S4.** Hazard ratios (HR) and 95% confidence intervals (CI) from multivariate Cox proportional hazards models for all-cause mortality according to categories of body mass index among African Americans study participants without chronic illness^a^ at baseline who never smoked, stratified by region of the country.

|  | **Region of US** | | | | | | | | | | | |
| --- | --- | --- | --- | --- | --- | --- | --- | --- | --- | --- | --- | --- |
|  | **Northeast** | | | **South** | | | **Midwest** | | | **West** | | |
|  | **HR** | **95% CI** | | **HR** | **95% CI** | | **HR** | **95% CI** | | **HR** | **95% CI** | |
| **BMI (kg/m^2^)** |  |  |  |  |  |  |  |  |  |  |  |  |
| 15-18.4 | 1.16 | (0.68- | 1.98) | 1.10 | (0.85- | 1.42) | 1.61 | (1.06- | 2.44) | 1.50 | (1.02- | 2.21) |
| 18.5-19.9 | 1.06 | (0.72- | 1.57) | 1.36 | (1.12- | 1.63) | 1.63 | (1.22- | 2.18) | 1.08 | (0.79- | 1.47) |
| 20-22.4 | 0.91 | (0.71- | 1.17) | 1.04 | (0.93- | 1.16) | 0.98 | (0.81- | 1.20) | 1.24 | (1.05- | 1.47) |
| 22.5-24.9 | 1.0 | Ref |  | 1.0 | Ref |  | 1.0 | Ref |  | 1.0 | Ref |  |
| 25-27.4 | 1.02 | (0.85- | 1.21) | 1.04 | (0.96- | 1.13) | 1.07 | (0.92- | 1.24) | 1.03 | (0.90- | 1.17) |
| 27.5-29.9 | 0.90 | (0.74- | 1.10) | 1.09 | (1.00- | 1.19) | 1.15 | (0.98- | 1.35) | 1.23 | (1.07- | 1.42) |
| 30-34.9 | 1.11 | (0.92- | 1.34) | 1.22 | (1.12- | 1.33) | 1.27 | (1.09- | 1.49) | 1.42 | (1.24- | 1.63) |
| 35-39.9 | 1.73 | (1.36- | 2.19) | 1.44 | (1.28- | 1.62) | 1.75 | (1.40- | 2.18) | 1.62 | (1.35- | 1.93) |
| 40-60 | 1.79 | (1.34- | 2.40) | 1.73 | (1.51- | 1.99) | 1.66 | (1.25- | 2.20) | 2.09 | (1.69- | 2.58) |

^a^ Chronic illness includes heart disease, stroke, or cancer (except non-melanoma skin cancer)

NOTE: Model adjusted for sex, education, marital status, alcohol consumption, and physical activity. Regions based on US Census divisions: http://www.census.gov/geo/www/us_regdiv.pdf
