# Supplementary material for: A Pooled Analysis of Body Mass Index and Mortality among African Americans
Source: PLoS One. 2014 Nov 17;9(11):e111980. doi: 10.1371/journal.pone.0111980 (PMC4234271; doi:10.1371/journal.pone.0111980)
Supplement: Table S5 — Hazard ratios (HR) and 95% confidence intervals (CI) from multivariate Cox proportional hazards models for all-cause mortality according to categories of body mass index among African American participants without chronic illness at baseline who never smoked, stratified by age at baseline. (DOCX) [file pone.0111980.s006.docx]

**Table S5.** Hazard ratios (HR) and 95% confidence intervals (CI) from multivariate Cox proportional hazards models for all-cause mortality according to categories of body mass index among African American participants without chronic illness at baseline who never smoked, stratified by age at baseline.

|  | **Age at baseline** | | | | | | | | | | | | | | |
| --- | --- | --- | --- | --- | --- | --- | --- | --- | --- | --- | --- | --- | --- | --- | --- |
|  | **<40** | | | **40-49** | | | **50-59** | | | **60-69** | | | **70+** | | |
|  | **HR** | **95% CI** | | **HR** | **95% CI** | | **HR** | **95% CI** | | **HR** | **95% CI** | | **HR** | **95% CI** | |
| **BMI (kg/m^2^)** |  |  |  |  |  |  |  |  |  |  |  |  |  |  |  |
| 15-18.4 | 1.56 | (0.84- | 2.88) | 1.71 | (0.93- | 3.15) | 1.09 | (0.66- | 1.80) | 1.56 | (1.11- | 2.20) | 1.05 | (0.80- | 1.39) |
| 18.5-19.9 | 0.89 | (0.52- | 1.53) | 1.32 | (0.87- | 2.00) | 2.08 | (1.56- | 2.76) | 1.11 | (0.86- | 1.44) | 1.32 | (1.07- | 1.64) |
| 20-22.4 | 1.10 | (0.78- | 1.53) | 0.98 | (0.77- | 1.24) | 0.96 | 0.80- | 1.16) | 1.06 | (0.92- | 1.21) | 1.10 | (0.96- | 1.26) |
| 22.5-24.9 | 1.0 | Ref |  | 1.0 | Ref |  | 1.0 | Ref |  | 1.0 | Ref |  | 1.0 | Ref |  |
| 25-27.4 | 1.17 | (0.84- | 1.62) | 1.07 | (0.89- | 1.29) | 1.05 | (0.93- | 1.20) | 1.03 | (0.94- | 1.14) | 1.01 | (0.90- | 1.13) |
| 27.5-29.9 | 1.26 | (0.87- | 1.83) | 1.10 | (0.90- | 1.35) | 1.18 | (1.04- | 1.36) | 1.13 | (1.02- | 1.25) | 1.00 | (0.88- | 1.13) |
| 30-34.9 | 1.42 | (1.02- | 1.97) | 1.21 | (1.00- | 1.46) | 1.49 | (1.31- | 1.69) | 1.22 | (1.10- | 1.35) | 1.05 | (0.93- | 1.19) |
| 35-39.9 | 1.65 | (1.10- | 2.46) | 1.36 | (1.07- | 1.73) | 1.78 | (1.51- | 2.09) | 1.54 | (1.34- | 1.78) | 1.36 | (1.13- | 1.64) |
| 40-60 | 2.02 | (1.35- | 3.03) | 1.86 | (1.46- | 2.37) | 2.09 | (1.73- | 2.53) | 1.73 | (1.45- | 2.06) | 1.19 | (0.90- | 1.58) |

^a^ Chronic illness includes heart disease, stroke, or cancer (except non-melanoma skin cancer)

NOTE: Model adjusted for sex, education, marital status, alcohol consumption, and physical activity
