# Supplementary material for: A Pooled Analysis of Body Mass Index and Mortality among African Americans
Source: PLoS One. 2014 Nov 17;9(11):e111980. doi: 10.1371/journal.pone.0111980 (PMC4234271; doi:10.1371/journal.pone.0111980)
Supplement: Table S6 — Hazard ratios (HR) and 95% confidence intervals (CI) from multivariate Cox proportional hazards models for all-cause mortality according to categories of body mass index among African American participants without chronic illness at baseline who never smoked, stratified by physical activity. (DOCX) [file pone.0111980.s007.docx]

**Table S6.** Hazard ratios (HR) and 95% confidence intervals (CI) from multivariate Cox proportional hazards models for all-cause mortality according to categories of body mass index among African American participants without chronic illness^a^ at baseline who never smoked, stratified by physical activity.

|  | **Physical Activity Level** | | | | | | | | |
| --- | --- | --- | --- | --- | --- | --- | --- | --- | --- |
|  | **Low** | | | **Medium** | | | **High** | | |
|  | **HR** | **95% CI** | | **HR** | **95% CI** | | **HR** | **95% CI** | |
| **BMI (kg/m^2^)** |  |  |  |  |  |  |  |  |  |
| 15-18.4 | 1.39 | (1.03- | 1.88) | 1.11 | (0.86- | 1.44) | 1.43 | (0.90- | 2.28) |
| 18.5-19.9 | 1.64 | (1.31- | 2.04) | 1.18 | (0.99- | 1.42) | 1.08 | (0.74- | 1.58) |
| 20-22.4 | 1.19 | (1.03- | 1.39) | 0.98 | (0.88- | 1.09) | 1.09 | (0.89- | 1.33) |
| 22.5-24.9 | 1.0 | Ref |  | 1.0 | Ref |  | 1.0 | Ref |  |
| 25-27.4 | 1.00 | (0.89- | 1.13) | 1.04 | (0.96- | 1.12) | 1.17 | (1.00- | 1.37) |
| 27.5-29.9 | 1.09 | (0.96- | 1.23) | 1.10 | (1.01- | 1.20) | 1.25 | (1.06- | 1.49) |
| 30-34.9 | 1.21 | (1.08- | 1.36) | 1.24 | (1.14- | 1.35) | 1.49 | (1.27- | 1.75) |
| 35-39.9 | 1.46 | (1.27- | 1.68) | 1.61 | (1.43- | 1.82) | 1.70 | (1.33- | 2.15) |
| 40-60 | 1.66 | (1.43- | 1.93) | 1.97 | (1.68- | 2.30) | 2.18 | (1.59- | 2.99) |

^a^ Chronic illness includes heart disease, stroke, or cancer (except non-melanoma skin cancer)

NOTE: Model adjusted for sex, education, marital status, and alcohol consumption.
