# Supplementary material for: A Pooled Analysis of Body Mass Index and Mortality among African Americans
Source: PLoS One. 2014 Nov 17;9(11):e111980. doi: 10.1371/journal.pone.0111980 (PMC4234271; doi:10.1371/journal.pone.0111980)
Supplement: Table S7 — Hazard ratios (HR) and 95% confidence intervals (CI) from multivariate Cox proportional hazards models for all-cause mortality according to categories of body mass index among African American participants without chronic illness at baseline who never smoked, stratified by specific cause of death and gender. (DOCX) [file pone.0111980.s008.docx]

**Table S7.** Hazard ratios (HR) and 95% confidence intervals (CI) from multivariate Cox proportional hazards models for all-cause mortality according to categories of body mass index among African American participants without chronic illness^a^ at baseline who never smoked, stratified by specific cause of death and gender.

| **MALES** | **Cause of Death** | | | | | | | | | | | |
| --- | --- | --- | --- | --- | --- | --- | --- | --- | --- | --- | --- | --- |
|  | **All Cancer** | | | | **All CVD** | | | | **All Other Non-External Causes ^b^** | | | |
|  | **Deaths** | **HR** | **95% CI** | | **Deaths** | **HR** | **95% CI** | | **Deaths** | **HR** | **95% CI** | |
| **BMI (kg/m^2^)** |  |  |  |  |  |  |  |  |  |  |  |  |
| 15-18.4 | 10 | 1.79 | (0.94- | 3.40) | 8 | 0.95 | (0.47- | 1.92) | 10 | 1.83 | (0.96- | 3.49) |
| 18.5-19.9 | 12 | 1.40 | (0.78- | 2.53) | 24 | 1.57 | (1.02- | 2.41) | 22 | 2.12 | (1.34- | 3.35) |
| 20-22.4 | 68 | 1.08 | (0.82- | 1.43) | 102 | 1.02 | (0.81- | 1.29) | 80 | 1.24 | (0.95- | 1.62) |
| 22.5-24.9 | 176 | 1.0 | Ref |  | 268 | 1.0 | Ref |  | 170 | 1.0 | Ref |  |
| 25-27.4 | 245 | 0.97 | (0.80- | 1.17) | 416 | 1.09 | (0.93- | 1.27) | 233 | 0.96 | (0.79- | 1.17) |
| 27.5-29.9 | 166 | 0.94 | (0.76- | 1.17) | 307 | 1.17 | (0.99- | 1.37) | 166 | 1.00 | (0.81- | 1.24) |
| 30-34.9 | 148 | 1.10 | (0.89- | 1.38) | 291 | 1.48 | (1.25- | 1.75) | 171 | 1.36 | (1.10- | 1.68) |
| 35-39.9 | 36 | 1.32 | (0.92- | 1.90) | 70 | 1.78 | (1.36- | 2.32) | 43 | 1.62 | (1.16- | 2.28) |
| 40-60 | 7 | 0.92 | (0.43- | 1.97) | 24 | 2.06 | (1.35- | 3.15) | 25 | 3.04 | (1.97- | 4.68) |
| **FEMALES** | **Cause of Death** | | | | | | | | | | | |
|  | **All Cancer** | | | | **All CVD** | | | | **All Other Non-External Causes ^b^** | | | |
|  | **Deaths** | **HR** | **95% CI** | | **Deaths** | **HR** | **95% CI** | | **Deaths** | **HR** | **95% CI** | |
| **BMI (kg/m^2^)** |  |  |  |  |  |  |  |  |  |  |  |  |
| 15-18.4 | 17 | 0.89 | (0.55- | 1.45) | 42 | 1.32 | (0.96- | 1.82) | 34 | 1.23 | (0.87- | 1.76) |
| 18.5-19.9 | 41 | 0.99 | (0.71- | 1.36) | 71 | 1.27 | (0.99- | 1.63) | 73 | 1.42 | (1.11- | 1.83) |
| 20-22.4 | 192 | 1.03 | (0.86- | 1.23) | 227 | 0.98 | (0.83- | 1.15) | 213 | 1.02 | (0.86- | 1.21) |
| 22.5-24.9 | 349 | 1.0 | Ref |  | 435 | 1.0 | Ref |  | 380 | 1.0 | Ref |  |
| 25-27.4 | 445 | 1.04 | (0.90- | 1.19) | 628 | 1.16 | (1.03- | 1.32) | 455 | 0.98 | (0.86- | 1.12) |
| 27.5-29.9 | 359 | 1.16 | (1.00- | 1.34) | 512 | 1.30 | (1.14- | 1.48) | 319 | 0.96 | (0.83- | 1.11) |
| 30-34.9 | 469 | 1.17 | (1.01- | 1.34) | 700 | 1.44 | (1.27- | 1.62) | 457 | 1.08 | (0.94- | 1.24) |
| 35-39.9 | 165 | 1.20 | (0.99- | 1.45) | 281 | 1.99 | (1.71- | 2.32) | 193 | 1.48 | (1.24- | 1.77) |
| 40-60 | 105 | 1.28 | (1.03- | 1.60) | 190 | 2.38 | (2.00- | 2.83) | 147 | 1.88 | (1.54- | 2.28) |

^a^ Chronic illness includes heart disease, stroke, or cancer (except non-melanoma skin cancer)

^b^ Includes all causes of death except cancer, CVD, and external causes. Models adjusted for sex, education, marital status, alcohol consumption, and physical activity. Models stratified by cohort.
