# Supplementary material for: A Pooled Analysis of Body Mass Index and Mortality among African Americans
Source: PLoS One. 2014 Nov 17;9(11):e111980. doi: 10.1371/journal.pone.0111980 (PMC4234271; doi:10.1371/journal.pone.0111980)
Supplement: Table S8 — Impact of omitting each cohort from analysis on hazard ratios (HR) and 95% confidence intervals (CI) for all-cause mortality per 5-unit increase in body mass index (BMI) in healthy, never smokers. (DOCX) [file pone.0111980.s009.docx]

**Table S8.** Impact of omitting each cohort from analysis on hazard ratios (HR) and 95% confidence intervals (CI) for all-cause mortality per 5-unit increase in body mass index (BMI) in healthy, never smokers**.**

|  | **BMI 15-24.9 kg/m^2^** | | | **BMI 25-60 kg/m^2^** | | |
| --- | --- | --- | --- | --- | --- | --- |
| **Omitted Study** | **HR** | **(95% CI)** | | **HR** | **(95% CI)** | |
| **Males** |  |  | |  |  | |
| AARP | 0.75 | (0.62 - | 0.90) | 1.21 | (1.14 - | 1.28) |
| AHS2 | 0.75 | (0.63 - | 0.90) | 1.21 | (1.15 - | 1.28) |
| CPSII | 0.57 | (0.41 - | 0.78) | 1.28 | (1.19 - | 1.38) |
| MEC | 0.73 | (0.60 - | 0.88) | 1.17 | (1.11 - | 1.24) |
| PLCO | 0.73 | (0.61 - | 0.87) | 1.21 | (1.15 - | 1.28) |
| SCCS | 0.75 | (0.62 - | 0.90) | 1.23 | (1.16 - | 1.30) |
| **Females** |  |  |  |  |  |  |
| AARP | 0.91 | (0.82 - | 1.02) | 1.17 | (1.14 - | 1.20) |
| AHS2 | 0.90 | (0.81 - | 1.01) | 1.18 | (1.15 - | 1.21) |
| BWHS | 0.91 | (0.81 - | 1.02) | 1.18 | (1.15 - | 1.21) |
| CPSII | 0.68 | (0.56 - | 0.83) | 1.18 | (1.14 - | 1.23) |
| MEC | 0.93 | (0.83 - | 1.04) | 1.17 | (1.14 - | 1.21) |
| PLCO | 0.90 | (0.81 - | 1.00) | 1.18 | (1.15 - | 1.21) |
| SCCS | 0.92 | (0.82 - | 1.03) | 1.19 | (1.16 - | 1.22) |

Note: Models stratified by BMI 15-24.9 and BMI 25-60 kg/m^2^. BMI analyzed as continuous measure in each stratified model. Models adjusted for education, marital status, alcohol consumption, and physical activity.
